# Radiation dose reduction in CT-guided periradicular injections in lumbar spine: Feasibility of a new institutional protocol for improved patient safety

**DOI:** 10.1186/1754-9493-6-19

**Published:** 2012-08-13

**Authors:** Juraj Artner, Balkan Cakir, Sebastian Weckbach, Heiko Reichel, Friederike Lattig

**Affiliations:** 1 Dept. of Orthopaedic Surgery, University of Ulm, RKU, 89081 Ulm, Germany; 2 Dept. of Trauma-, Hand-, Plastic- and Reconstructive Surgery, University of Ulm, 89075 Ulm, Germany

**Keywords:** CT-Guided spinal injections, Periradicular injection, Computed tomography, Lumbar spine, Low dose protocol

## Abstract

**Background:**

Image guided spinal injections are successfully used in the management of low back pain and sciatica. The main benefit of CT-guided injections is the safe, fast and precise needle placement, but the radiation exposure remains a serious concern. The purpose of the study was to test a new institutional low-dose protocol for CT-guided periradicular injections in lumbar spine to reduce radiation exposure while increasing accuracy and safety for the patients.

**Methods:**

We performed a retrospective analysis of a prospective database during a 4-month period (Oct-Dec 2011) at a German University hospital using a newly established low-dose-CT-protocol for periradicular injections in patients suffering from lumbar disc herniation and nerve root entrapment. Inclusion criteria were acute or chronic nerve root irritation due to lumbar disc hernia, age over 18, compliance and informed consent. Excluded were patients suffering from severe obesity (BMI > 30), coagulopathy, allergy to injected substances, infection and non-compliant patients. Outcome parameters consisted of the measured dose length product (mGycm2), the amount of scans, age, gender, BMI and the peri-interventional complications. The results were compared to 50 patients, treated in the standard-interventional CT-protocol for spinal injections, performed in June-Oct 2011, who met the above mentioned inclusion criteria.

**Results:**

A total amount of 100 patients were enrolled in the study. A significant radiation dose reduction (average 85.31%) was achieved using the institutional low-dose protocol compared to standard intervention mode in CT-guided periradicular injections in lumbar spine. Using the low-dose protocol did not increase the complications rate in the analyzed cohort.

**Conclusions:**

Low-dose-CT-protocols for lumbar perineural injections significantly reduce the exposure to radiation of non-obese patients without an increase of complications. This increases long-time patient safety of stochastic radiation effects.

## Background

Spinal injections are an important tool in the diagnosis and therapy of spinal disorders. Periradicular injections, also termed nerve root-blocks or perineural injections, are used despite their controversial evidence successfully by orthopaedic surgeons, radiologists, neurosurgeons and pain interventionalists for decades in the diagnosis and therapy of nerve root irritation due to intervertebral disc herniation, zygapophysial joint hypertrophy and spondylolisthesis [1,2]. The performing interventionalists are using different types of imaging for a safe injection guidance, ie. ultrasound, MRI, fluoroscopy or computed tomography. The main benefit of CT-guided spinal injections is the precise and safe needle placement, especially in patients with severe degenerative spinal changes. Despite the safety and precision of image-guided interventions, the radiation exposure of the patient and interventionalist still remains a serious concern [3-8]. The stochastic radiation effect due to a cumulative radiation dose in patients suffering from degenerative spinal disorders implicating a long-term therapy and use of spinal injections is often used as an argument against the use of CT-guidance [9]. The aim of the following article was to test a new institutional low-dose-protocol for CT-guided periradicular injections in lumbar spine to minimize the radiation exposure and increase patient`s safety.

## Methods

A retrospective analysis of a prospective database during a 4-month period (Oct-Dec 2011) was performed in a German University hospital setting using a newly established institutional low-dose-CT-protocol for periradicular injections. Included were adult inpatients (> 18 years), suffering of radicular pain due to a lumbar disc herniation. Excluded from the study were patients, who did not sign an informed consent, had an absolute indication for surgery, incompliant patients, patients suffering from severe obesity (BMI > 30), coagulopathy, gravidity, infection or severe osteoporosis. CT-guided periradicular injections were performed by one experienced interventionalist, using the SOMATOM Emotion CT-scanner (syngoCT 2009E, 16-slice solution, Siemens Medical Solutions AG, Erlangen, Germany) in low-dose mode, consisting of a narrowing of the scanned area and a reduction of energy and tube current (for details see Appendix 1: technical description). Outcome parameters consisted of the measured radiation dose (DLP, dose length product in mGycm2), the segment, the amount of scans in the intervention mode, age, gender, BMI and peri-interventional complications. The results were compared to 50 patients, treated in the standard-interventional CT-protocol for spinal injections, performed in June-Oct 2011, who met the above mentioned inclusion and exclusion criteria. Statistical analysis was performed using the software SPSS Statistics (ver. 17.0, IBM, Armonk, New York, 2008). The statistical significance of the difference between the groups was calculated with the Mann–Whitney-*U*-test. A p-value < 0.05 was considered statistically significant.

## Results

**Figure 1:**
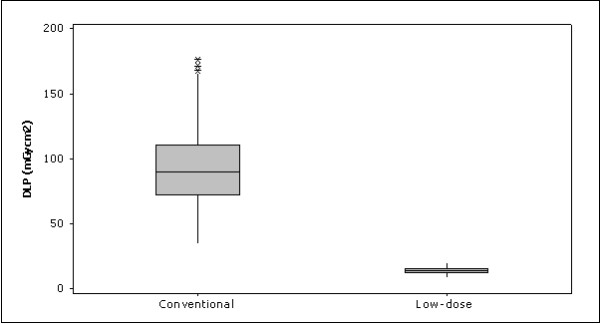
Comparison of the conventional and low-dose lumbar periradicular injection doses.

A total amount of 50 perineural injections were performed in the low-dose protocol at the lumbar spine. Compared to 50 perineural injections performed in the conventional interventional CT-protocol, an average dose reduction of 85.31% could be achieved using the low-dose protocol (see Figure 1 and Table 1). We calculated a mean DLP of 13.87 mGycm2 (SD = 2.48) for the low-dose CT-protocol cohort (mean age = 57.46 years, range 21–87; 27 females, 23 males; mean BMI = 25.52) and 94.44 mGycm2 (SD = 33.34) for the conventional CT-protocol cohort (mean age = 55.29 years, range 19–88; 31 females, 19 males; mean BMI = 25.58). A total amount of 3–6 scans (median = 4) was used in both intervention modes. There was a correct needle placement in all interventions. No complications were registered during or after the procedures. 

**Table 1:** Distribution of CT-guided periradicular injections in conventional and low-dose mode with radiation dose comparisons

**Nerve root**	**Mode**	**N**	**DLP (mGycm2) mean dose**	**DLPSD**	**M-W*u*-test**	**Dose reduction %**
**L 2**	Conventional m.	4	78.00	22.21		
	Low-dose mode	4	13.91	1.78	*p = 0.0304*	- 82.17%
**L 3**	Conventional m.	11	90.09	42.67		
	Low-dose mode	4	12.37	2.54	*p = 0.0050*	- 86.27%
**L 4**	Conventional m.	10	86.60	22.43		
	Low-dose mode	11	13.30	2.62	*p < 0.001*	- 84,64%
**L 5**	Conventional m.	16	103.19	34.70		
	Low-dose mode	22	14.36	2.68	*p < 0.001*	- 86.08%
**S 1**	Conventional m.	9	100.22	33.07		
	Low-dose mode	9	14.03	2.10	*p < 0.001*	- 85.67%
**Total L 1 - S 1**	Conventional m.	50	94.44	33.34		
	Low-dose mode	50	13.87	2.48	*p < 0.001*	- 85.31%

Abbreviations: DLP = dose length product (mGycm2), SD = standard deviation, N = amount, M-W = Mann–Whitney (U-) test.

## Discussion

The aim of the study was to test our new institutional low dose protocol for CT-guided selective nerve root blocks on lumbar spine, consisting of the steps: reducing the tube current and energy in topogram acquisition, narrowing the scanned area of interest and reducing the energy and tube current in the interventional mode. A significant dose reduction (mean 85.31%) could be achieved in all lumbar segments without increased complications in non-obese patients. The low-dose protocol presented a lower image quality (contrast), but a good visibility even of the thinnest needle caliber used (29 Gauge needles were used in all subjects) was still maintained in all procedures.

CT-guidance improves precision and safety in spinal injections. Regarding the visualization and a longer lasting effect, CT-guided periradicular injections are described to be superior to fluoroscopy-guided injections [10], but the exposure to radiation which may vary significantly between and within performing institutions still remains a serious concern [11,12]. The cumulative risk of stochastic radiation effects when performing repetitive injections in patients with degenerative spinal disorders is underestimated. In the analyzed low-dose cohort we calculated a mean radiation dose of 13.87 mGycm2, corresponding to an effective radiation dose of 0.21 mSv for interventions on the trunk. Similar results in dose reductions with other settings were described for spinal injections by [Schmid et al. 13] on standard Alderson Rando phantoms . The authors calculated for CT-guided epidural injections radiation doses ranging between 1.51-3.53 mSv in standard intervention mode and 0.22-0.43 mSv for a low-dose protocol. Most reports in the literature describe higher radiation doses for CT-guided spinal injections, ranging from 3.3 to 9.1 mSv (corresponding to 220–606 mGycm2) [14-16]. Hoang et al. described average radiation doses of 3.35 mSv for epidural injections using CT-fluoroscopy-guidance [16]. Shepherd et al. calculated similar mean effective radiation dose of 3.3 mSv for lumbar spinal injections [15]. For other CT-guided interventions, the doses reported in literature may range up to 830 mGy (corresponding to 12.45 mSv) [17]. The mean effective radiation doses for CT-guided spinal injections in our department are lower than all the above mentioned doses even in the standard intervention mode, ranging between 1.30-1.44 mSv in lumbar perineural and epidural injections [18]. Using the new low-dose protocol for periradicular injections, these doses could be once more reduced significantly. This underlines the need of further dose reduction and standardization of settings.

Our study has limitations. Overweight patients were included, but severe obesity (BMI 30 or more) was set as an exclusion criterion. The image quality is reduced and may cause problems in overweight patients. The dose reduction in perineural injections was also possible in patients suffering from overweight, because the needle and the osseous landmarks of the neuroforamen were visible even in the reduced image quality. We conclude that this point may become problematic in CT-guided epidural injections, where the visibility of soft tissues like Lig.flavum and dural sac may be the limiting factor for dose reduction in patients with higher BMI (scatter radiation). In such cases, use of larger needle calibers, the “loss of resistance”-technique and contrast solution injection may be helpful.

## Conclusions

Significant reduction of radiation dose can be achieved in periradicular injections in lumbar spine without a reduction of precision and safety for the patient. Working with low-dose protocols requires only a short learning curve and is practicable on most CT-scanners. Reducing the cumulative radiation exposure in patients with spinal disorders undergoing CT-guided injections, using thin needle systems which reduce tissue traumatization as well as maintaining a good needle targeting using CT-guidance in low-dose protocols contribute to an increase of patient safety.

## Appendix 1: Technical considerations

The common CT-guided spinal interventions consist of the following steps

1- Topogram acquisition in sagittal plane

2- Selection of the area of interest (ARI)

3- CT-scan in transversal plane

4- Biopsy mode for interventions

**Figure 2:**
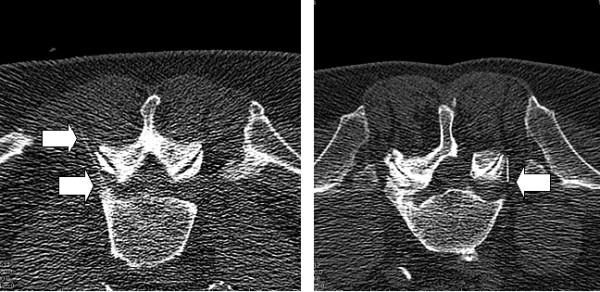
**CT-guided nerve root L5- injections.** CT-guided periradicular injections at the nerve root L5 in low-dose mode in two male patients. Note the acceptable visibility of the needle tip (arrow) in both interventions, even in reduced contrast. The right image presents a status post hemilaminectomy.

Significant dose reduction can be achieved by modifications in all steps mentioned above [19,20]. The following technical considerations demonstrate the stepwise dose reduction. Note, that reductions in tube energy have to be set/ modified for all steps before the start of the intervention (step 1):**1.** The dose reduction should begin at this step, because already the topogram acquisition contributes to high radiation exposure. Keeping the topogram scan as small as necessary (manual stop) with reduced energy levels and tube current (80 kV and 100 mA) decreases the radiation dose. The simultaneous decrease of the image quality can be accepted without a loss of information. With the exception of severe obesity or osteoporosis, the area of interest - the neuroforamen can be identified even in lower quality images. **2.** It is also important to set the area of interest (ARI) as small as possible, because it determines the amount of scanned slices in step 3. The targeted area of periradicular injections is the neuroforamen, which is easy to identify on the topogram. To avoid a painful punction of the nerve root the upper parts of the neuroforamen should be targeted. Placing the ARI-box on the superior border of the neuroforamen and narrowing the height to a minimum reduces the scanned ARI to 2–4 scans. **3.** In the next step, the ARI is scanned (reduced to 80 kV and 80 mA) and transversal slices of the related segment are acquired. The image quality is reduced due to dose reduction, but the target area between the bony landmarks of the neuroforamen which have a good contrast (posterior aspect of the vertebral body and zygapophysial joints) is still good to identify. The approach for the punction is planned for an oblique direction of the needle. **4.** In the final step an introducer-needle is placed in the planned direction and a 29-G-needle is advanced onto the neuroforamen, controlled by stepwise scans in biopsy mode (reduced to 80 kV and 50 mA). Despite the reduction of image quality, even the thin 29-G-needle is easy to identify and the needle tip always visible in dose reduced scans (see Figure 2), and the medications (analgesics with or without corticosteroid) can be injected. There is no need for use of contrast solutions in extraforaminal periradicular injections. Patients are monitored at least for 30 min after the procedures.

## Competing interests

The authors declare that they have no competing interests.

## Authors’ contributions

JA, BC, SW, HR, FL designed the study. JA developed the low-dose protocol and was the performing interventionalist. All authors contributed and approved the final version of the manuscript.
